# Non-targeted metabolomics of cooked cowpea *(Vigna unguiculata)* and pigeon pea *(Cajanus cajan)* from Ghana using two distinct and complementary analytical platforms

**DOI:** 10.1016/j.fochms.2022.100087

**Published:** 2022-02-14

**Authors:** Brooke Sayre-Chavez, Bridget Baxter, Corey D. Broeckling, María Muñoz-Amatriaín, Mark Manary, Elizabeth P. Ryan

**Affiliations:** a Department of Soil and Crop Sciences, Colorado State University, Fort Collins, CO 80523, USA; b Department of Environmental and Radiological Health Sciences, Colorado State University, Fort Collins, CO 80523, USA; c Analytical Resources Core: Bioanalysis and Omics Center, Colorado State University, Fort Collins, CO 80523, USA; d Department of Molecular Biology, University of León, 24071 León, Spain; e Department of Pediatrics, Washington University School of Medicine in St. Louis, St. Louis, MO 63110, USA

**Keywords:** Pipecolic acid (PubChem CID: 849), 3-(all-*trans*-nonaprenyl)benzene-1,2-diol (PubChem CID: 25245248), N-tetracosanoylphytosphingosine (PubChem CID: 14653935), Sitoindoside II (PubChem CID: 131751526), Tonkinelin (PubChem CID: 73088078), Pheophytin A (PubChem CID: 135398712), Linoleoyl ethanolamide (PubChem CID: 5283446), Piptamine (PubChem CID: 10664275), *Vigna unguiculata*, *Cajanus cajan*, *Phaseolus vulgaris*, Non-targeted metabolomics, UPLC-MS, Legumes, Phytochemicals

## Abstract

Legumes are global staple foods with multiple human health properties that merit detailed composition analysis in cooked forms. This study analyzed cowpea [*Vigna unguiculata*] (three varieties: Dagbantuya, Sangyi, and Tukara), pigeon pea [*Cajanus cajan*], and common bean [*Phaseolus vulgaris*] using two distinct ultra-performance liquid chromatography mass spectrometry (UPLC-MS) platforms and analytical workflows. Comparisons between cowpea and pigeon pea consumed in Ghana, and common bean (navy bean) from USA, revealed 75 metabolites that differentiated cowpeas. Metabolite fold-change comparisons resulted in 142 metabolites with significantly higher abundance in cowpea, and 154 higher in abundance from pigeon pea. 3-(all-*trans*-nonaprenyl)benzene-1,2-diol, N-tetracosanoylphytosphingosine, and sitoindoside II are novel identifications in cowpea, with notably higher abundance than other legumes tested. Cowpea variety specific markers were tonkinelin (Dagbantuya), pheophytin A (Sangyi), and linoleoyl ethanolamide (Tukara). This study identified novel cowpea and pigeon pea food metabolites that warrant continued investigation as bioactive food components following consumption in people.

## Highlights

• Cowpea varieties represent diverse staple foods in Sub-Saharan Africa.
• Cowpea metabolite composition is different from pigeon pea and common bean.
• Cowpea metabolites included tonkinelin, pheophytin A, and linoleoyl ethanolamide.
• Pheophytin A was only detected in the cowpea variety Sangyi.
• Pipecolic acid identification was confirmed for all three legumes.

## Graphical abstract

**Figure:**
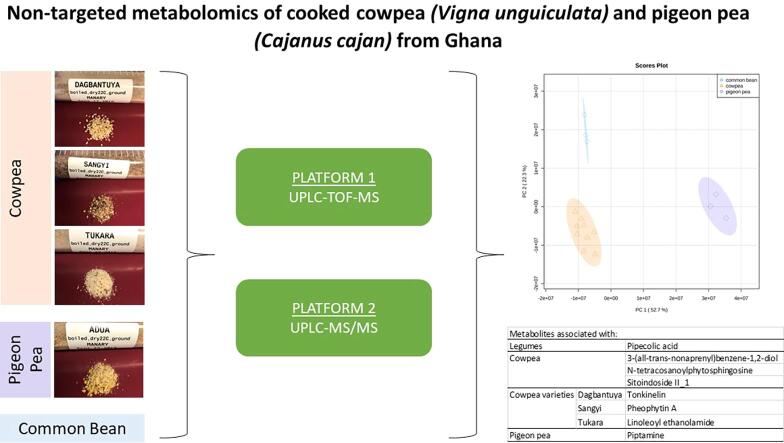

## 1 Introduction

Cowpea (*Vigna unguiculata*) is a warm-season and nitrogen-fixing legume well adapted to the sandy soils and low-input farming practices of sub-Saharan Africa (Ji et al., 2019, Vaillancourt and Weeden, 1992). Cowpea is a nutritious food due to high protein content, and abundance of vitamins, trace minerals, antioxidants, amino acids, fibers, lipids, and phytochemicals that have bioactivity and human health properties (Abizari et al., 2013, Awika and Duodu, 2017, Jayathilake et al., 2018). In addition to containing nutrients that can combat child malnutrition (Stephenson et al., 2017), consumption of cowpeas is associated with lowered risk of high cholesterol and blood pressure, as well as a reduced risk of developing diseases such as diabetes and cancer (Awika and Duodu, 2017, Jayathilake et al., 2018). Cowpea is highly valued as a cash crop and used for animal feed to support livestock production that bolster farmers income. In Ghana, consumers will pay a premium for certain cowpea varieties, which provides the incentive to sell the food crop (Langyintuo et al., 2003). Detailed compositional analysis of cowpea varieties in the cooked forms that are consumed by people is lacking.

Pigeon pea (*Cajanus cajan*) is another important nitrogen-fixing, warm season legume grown in sub-Saharan Africa (Adjei-Nsiah, 2012). Like cowpea, it is drought tolerant and well adapted to low-input farming practices (Saxena, 2008). Pigeon pea is often used as a border crop or grown in an intercropping system to help improve soil fertility, a major factor with implications on local food security (Abunyewa & Karbo, 2005). Pigeon pea is also valued for human and animal consumption due to its high protein content (Abunyewa and Karbo, 2005, Adjei-Nsiah, 2012, Nwokolo, 1987, Pal et al., 2011, Saxena, 2008). Pigeon pea is rich in nutrients, and therefore also relevant for helping to alleviate malnutrition (Nwokolo, 1987, Pal et al., 2011). Based on anecdotal evidence of a local focus group in Ghana (described in section 2.1), pigeon peas were classified as integral part of the local diet. Cowpea and pigeon pea are consumed in multiple ways as a whole bean (boiled), and then may be fried, dried, steamed as a paste, or made into flour forms (Langyintuo et al., 2003).

Soybeans, various dry beans, green peas, chickpeas, and lentils have been investigated for identification of small molecule markers in the food and following human dietary exposure (Borresen et al., 2017, Lu et al., 2010, Madrid-Gambin et al., 2017, Perera et al., 2015, Sri Harsha et al., 2018, Zarei et al., 2021). For example, pipecolic acid and s-methylcysteine have been quantified in legumes while also being proposed as biomarkers following common dry bean consumption (Perera et al., 2015). A series of amino acids, lipids, peptides and xenobiotics were reported for cowpea from a single Malawi study, including ophthalmate, palmitoyl-oleoyl-glycerol (16:0/18:1), alanylleucine, and benzoate (Borresen et al., 2017). This study compared a single cowpea to dry bean and soybean blends using a single metabolomics platform, but this study did not compare cowpea varieties. The analysis of food metabolomes for cowpea and pigeon pea varieties that are consumed locally in Ghana was a major objective herein for enhancing linkages to maternal and child nutritional studies with these foods, and particularly in undernourished populations. To our knowledge, there are no studies specifically aimed to identify dietary biomarkers in pigeon pea. In addition to human health applications, these cowpea and pigeon pea cooked food metabolite profiles will have utility in breeding programs geared towards improving nutritional quality traits.

In this study, we analyzed the metabolite profiles of three different cowpea varieties commonly consumed in Ghana with comparisons to pigeon pea that is also consumed in combination with cowpeas. A common bean of the Navy market class from the USA was used as a reference legume that has already been characterized via metabolomics (Perera et al., 2015, Zarei et al., 2021). The main objective was to examine and compare the metabolite composition of cowpeas and pigeon pea types consumed in Ghana using two distinct and complementary non-targeted metabolomics workflows as a novel, comprehensive chemical profiling approach.

## 2 Materials and methods

### 2.1 Cooked legume flours

Four varieties of local “cowpea” flours (Dagbantuya, Sangyi, Tukara, and Adua), identified by a local community focus group in the region, were collected from a local market in Tamale (northern Ghana). A local legume expert from the Savanna Agricultural Research Institute accompanied the investigator to the general market in Tamale, Ghana. There he identified the four types by sight and familiarity. 10 kg amounts of each type were purchased from multiple vendors, such that 100kg total of each type were acquired. These were thoroughly mechanically mixed homogenizing them by type. All samples were cooked, dried, and milled into flours for metabolite analysis. Seed morphology together with metabolomics data (see Section 3.1.2) revealed that one of the varieties (Adua) was a pigeon pea. The common bean flour (Navy market class) was purchased from ADM Edible Bean Specialties, Inc. (Archer Daniels Midland Company, Decatur, Illinois, USA).

Flours were prepared by boiling the legumes for 45 min., draining them, and then drying them on a flat sheet in an oven at 40 °C. The dried, cooked legumes were then ground to a fine powder with a mortar and pestle. Flours were stored in sealed conical tubes until the time of analyses.

### 2.2 Metabolomics Platform 1: CSU analytical resources core – Bioanalysis and omics laboratory (ARC-BIO) (Fort Collins, CO, USA)

#### 2.2.1 Sample preparation

For each legume sample, 50 (+/-1) mg of each cooked legume flour was weighed into a 2.0 mL eppendorf tube (Cole-Parmer, Vernon Hills, IL, USA, #06335–02) with 1.5 mL of absolute methanol (Thermo Scientific, Waltham, MA, USA, Fisher Optima LC-MS grade, A4564). Three process blanks were prepared alongside the legume samples, where solvent was used to extract from empty tubes. Samples were vortex mixed and extracted with shaking for one hour at 4 °C. After centrifugation at 4 °C, 13,000 xg, 1.0 mL of supernatant was collected and transferred to an autosampler vial (VWR, Radnor, PA, USA, #66009-854). 100 uL of supernatant was collected from each sample to generate a pooled QC. Sample processing order was randomized.

#### 2.2.2 Ultra performance liquid chromatography-time of flight mass spectroscopy (UPLC-TOF-MS)

Three microliters of legume flour sample extract were injected onto a Waters Acquity UPLC system (Waters Corporation, Milford, MA, USA) in randomized order with a pooled quality control (QC) injection after every 5 samples. Separation was achieved using a Waters Acquity UPLC CSH Phenyl Hexyl column (1.7 μM, 1.0 × 100 mm) (Waters Corporation, Milford, MA, USA), using a gradient from solvent A (Water, 2 mM ammonium formate) to solvent B (Acetonitrile, 0.1% formic acid). Injections were made in 99% A, held at 99% A for 1 min, ramped to 98% B over 12 min, held at 98% B for 3 min, and then returned to starting conditions over 0.05 min and allowed to re-equilibrate for 3.95 min, with a 200 μL/min constant flow rate. The column and samples were held at 65 °C and 6 °C, respectively. The column eluent was infused into a Waters Xevo G2-XS Q-TOF-MS (Waters Corporation, Milford, MA, USA) with an electrospray source in positive mode, scanning 50–1200 *m*/*z* at 0.1 s per scan, alternating between MS (6 V collision energy) and MSE mode (15–30 V ramp). Calibration was performed using sodium formate with 1 ppm mass accuracy. The capillary voltage was held at 700 V, source temperature at 140 °C, and nitrogen desolvation temperature at 600 °C with a desolvation gas flow rate of 1000 L/hr.

#### 2.2.3 Data normalization, filtration, and grouping

RAMClustR version 1.1.0 in R version 3.6.2 (2019-12-12)) was used to normalize, filter, and group features into spectra from XCMS output data (Smith et al., 2006, Tautenhahn et al., 2008). Features which failed to demonstrate signal intensity of at least 3-fold greater in QC samples than in blanks were removed from the feature dataset. 18,561 of 52,141 features were removed. Features with missing values were replaced with small values to simulate noise and then the minimum detected or simulated value was multiplied by 0.1. The filled value was the absolute value of this value. Features were normalized by linearly regressing run order versus qc feature intensities to account for instrument signal intensity drift. Only features with a regression *p*-value less than 0.05 and an r-squared greater than 0.1 were corrected. Features were filtered based on their qc sample CV values. Only features with CV values less than or equal to 0.3 in MS or MSMSdata sets were retained. 22,091 of 33,580 features were removed. Features were additionally normalized to total extracted ion signal to account for differences in total solute concentration. Features were clustered using the ramclustR algorithm. Parameter settings were as follows: st (sigma t, controlling retention time tolerance) = 2.22, sr = (sigma r, controlling correlational strength tolerance) 0.7, maxt (beyond which similarity is not calculated) = 222, deepSplit (controlling tree cutting) = FALSE, hmax (maximum branch height) = 0.3, minModuleSize (number of features required per compound) = 2, and cor.method = pearson. Charge state detection was performed using the assign.z function using parameters: chargestate (maximum charge state) = 3, mzError = 0.005, nEvents = 2, minPercentSignal = 10, and assume1 = TRUE, which enforces a charge state = 1 when isotope-based inference is unclear. Molecular weight was inferred from in-source spectra (Broeckling et al., 2016) using the do.findmain function, which calls the interpretMSSpectrum package (Jaeger, Hoffman, Schmitt, & Lisec, 2016). Parameters for do.findmain were set to: mode = positive, mzabs.error = 0.002, ppm.error = 10, ads = default, scoring = auto, and use.z = TRUE, which ensures that *m*/*z* values are converted to explicit mass values having inferred charge state (z) above.

MSFinder (Tsugawa et al., 2016) was used for spectral matching, formula inference, and tentative structure assignment, and results were imported into the RAMClustR object. Annotations were assigned using the RAMClustR annotate function. Annotation priority was assigned from highest priority to lowest: MSFinder structure, MSFinder formula, interpretMSSpectrum M. Database priority was set to HMDB, PubChem, UNPD, ChEBI, PlantCyc, KNApSAcK, FooDB, DrugBank, LipidMAPS, and Urine. Compounds were assigned to chemical ontogenies using the ClassyFire API (Djoumbou, 2016).

### 2.3 MetaboAnalyst and statistical analysis

The normalized spectral abundance data was grouped by legume type and comparison grouping prior to input into MetaboAnalyst Version 5.0 (https://www.metaboanalyst.ca/), where the following statistical functions were performed; one-way ANOVA, dendrogram, heatmap, and fold-change analysis. Data was not additionally filtered, normalized, or transformed. ANOVA *p*-value cutoff was set to 0.05 and Fisher’s least significant difference (LSD) post-hoc analysis was used. Dendrograms used Euclidean distances and Ward clustering. Correlation heatmaps mapped the features with Pearson r distances. Fold change threshold was set to 2.

### 2.4 Metabolomics Platform 2: Metabolon, Inc. (Durham, NC, USA)

The samples of cowpea and pigeon pea flours were also sent to Metabolon, Inc. (Durham, NC, USA) for a comprehensive varietal analysis of the cowpea flours using 80% methanol extraction.

#### 2.4.1 Sample preparation

Samples were inventoried and accessioned into the Metabolon LIMS system where they were assigned a unique identifier and then stored at −80 °C until processed. Samples were prepared using the automated MicroLab STAR® system from Hamilton Company (Reno, NV, USA). Several recovery standards were added prior to the first step in the extraction process for QC purposes. To remove protein, dissociate small molecules bound to protein or trapped in the precipitated protein matrix, and to recover chemically diverse metabolites, proteins were precipitated with methanol under vigorous shaking for 2 min (Glen Mills GenoGrinder 2000 [Glen Mills Inc., Clifton, NJ, USA]) followed by centrifugation. The resulting extract was divided into four fractions: two for analysis by two separate reverse phase (RP)/UPLC-MS/MS methods with positive ion mode electrospray ionization (ESI), one for analysis by RP/UPLC-MS/MS with negative ion mode ESI, one for analysis by HILIC/UPLC-MS/MS with negative ion mode ESI. Samples were placed briefly on a TurboVap® (Zymark Corporation, Hopkinton, MA, USA) to remove the organic solvent. The sample extracts were stored overnight under nitrogen before preparation for analysis.

#### 2.4.2 Ultra performance liquid chromatography-tandem mass spectroscopy (UPLC-MS/MS)

All methods utilized a Waters ACQUITY ultra-performance liquid chromatography (UPLC) (Waters Corporation, Milford, MA, USA) and a Thermo Scientific Q-Exactive high resolution/accurate mass spectrometer (Thermo Fisher Scientific, Waltham, MA, USA) interfaced with a heated electrospray ionization (HESI-II) source and Orbitrap mass analyzer operated at 35,000 mass resolution. The sample extract was dried then reconstituted in solvents compatible to each of the four methods. Each reconstitution solvent contained d7-glucose (1.0 ng/mL–50.0 ng/mL); d3-leucine (0.25 ng/mL–5.0 ng/mL); d8-phenylalanine (0.25 ng/mL–3.0); d5-tryptophan (0.25 ng/mL–25.00 ng/mL); d5-hipppuric acid (0.25 ng/mL–25.0 ng/mL); Br-phenylalanine (0.25 ng/mL–3.0); d5-indole acetic acid (3.0 ng/mL–25.00 ng/mL); amitriptyline (0.5 ng/mL–3.0 ng/mL) and d9-progesterone (1.0 ng/mL–25.0 ng/mL) are included in the series of standards at fixed concentrations to ensure injection and chromatographic consistency (Evans et al., 2014). One aliquot (50 μL) was analyzed using acidic positive ion conditions, chromatographically optimized for more hydrophilic compounds. Supplementary Table 1 shows the gradient profiles for reverse phase and HILIC methods. In this method, the extract was gradient eluted from a C18 column (Waters UPLC BEH C18-2.1x100 mm, 1.7 µm [Waters Corporation, Milford, MA, USA]) using water and methanol, containing 0.05% perfluoropentanoic acid (PFPA) and 0.1% formic acid (FA). Another aliquot was also analyzed using acidic positive ion conditions, chromatographically optimized for more hydrophobic compounds. In this method, the extract was gradient eluted from the same afore mentioned C18 column using methanol, acetonitrile, water, 0.05% PFPA and 0.01% FA and was operated at an overall higher organic content. Another aliquot was analyzed using basic negative ion optimized conditions using a separate dedicated C18 column. The basic extracts were gradient eluted from the column using methanol and water, however with 6.5 mM Ammonium Bicarbonate at pH 8. The fourth aliquot was analyzed via negative ionization following elution from a HILIC column (Waters UPLC BEH Amide 2.1x150 mm, 1.7 µm [Waters Corporation, Milford, MA, USA]) using a gradient consisting of water and acetonitrile with 10 mM Ammonium Formate, pH 10.8. The MS analysis alternated between MS and data-dependent MSn scans using dynamic exclusion. The scan range varied slighted between methods but covered 70–1000 *m*/*z*.

#### 2.4.3 Data extraction and compound identification

Raw data was extracted, peak-identified and QC processed using Metabolon’s hardware and software. These systems are built on a web-service platform utilizing Microsoft’s .NET technologies, which run on high-performance application servers and fiber-channel storage arrays in clusters to provide active failover and load-balancing. Compounds were identified by comparison to library entries of purified standards or recurrent unknown entities. Metabolon maintains a library based on authenticated standards that contains the retention time/index (RI), mass to charge ratio (*m*/*z*), and chromatographic data (including MS/MS spectral data) on all molecules present in the library, which include more than 3300 commercially available purified standards. Furthermore, biochemical identifications are based on three criteria: retention index within a narrow RI window of the proposed identification, accurate mass match to the library +/- 10 ppm, and the MS/MS forward and reverse scores between the experimental data and authentic standards. Standard statistical analyses are performed in ArrayStudio on log transformed data.

## 3 Results

### 3.1 Identification of metabolites across legume types via non-targeted metabolomics

The CSU ARC-BIO metabolomics (Platform 1) yielded 775 metabolites across diverse classes. By superclass, there were 410 lipids and lipid-like molecules, 260 unclassified metabolites, 26 organic acids and derivatives, 18 organoheterocyclic compounds, 17 phenylpropanoids and polyketides, 14 organic oxygen compounds, 8 benzenoids, 8 hydrocarbons, 8 organic nitrogen compounds, 2 alkaloids and derivatives, 2 lignans/neolignans/related compounds, 1 nucleoside/nucleotide/analogue, and 1 organosulfur compound in the data (data available at: www.ebi.ac.uk/metabolights/MTBLS3619) (Haug et al., 2020).

The Metabolon Inc. analysis (Platform 2) yielded 441 metabolites in the cowpea and pigeon pea. There were 400 metabolites with known identifications categorized by super pathways. This included 134 lipids, 130 amino acids, 43 carbohydrates, 40 nucleotides, 24 cofactors/prosthetic groups/electron carriers, 21 secondary metabolites, 5 peptides, 2 xenobiotics, and 1 hormone (Supplementary Table 2).

#### 3.1.1 Metabolite profile comparisons across legume types revealed differences between cowpea, pigeon pea, and common bean

**Fig. 1:**
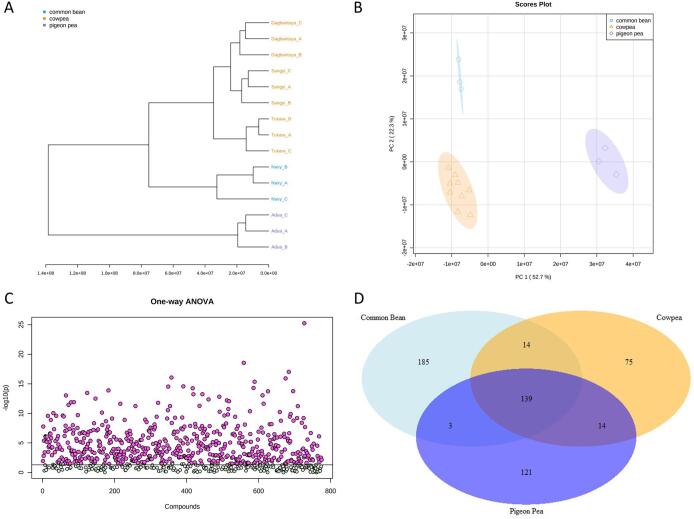
Legume metabolomes examined together by (A) Hierarchal cluster dendrogram based on Euclidean distance and Ward clustering. (B) Principal Component Analysis (PCA) scores plot. (C) One-way ANOVA plotting -log10(*p*-values) of all detected metabolites. Metabolites with significant differences in means across legume type are plotted in red (n = 551), metabolites with no significant difference in mean across legume types are plotted in green (n = 224). (D) Venn diagram indicating the number of metabolites that differentiate legume type based on Fisher’s least significant difference (LSD) post-hoc analysis.

Clear differences in small molecule profiles were observed between the legumes. Fig. 1(A and B) shows all legumes clustered by type (i.e., species). Principal component (PC) 1 explained 52.7% of the variation and mainly differentiated the pigeon pea (i.e., Adua) samples from cowpea and common bean, while PC2 explained 22.3% of the variation and separated the USA common bean samples from the Ghana cowpeas and pigeon pea (Fig. 1B). A dendrogram constructed using metabolite abundance profiles showed similar relationships between the three legumes (Fig. 1A). One-way ANOVA revealed 551 metabolites with significant differences in relative abundances between legume types (Fig. 1C). The most significant metabolites were TG(14:1(9Z)/14:1(9Z)/20:5(5Z,8Z,11Z,14Z,17Z)), PG(22:1(11Z)/22:1(11Z)), UNPD93557, C11H23N2O24PS10, and pipecolic acid, all of which have higher abundance in common bean compared to cowpea and pigeon pea (Supplementary Table 3). There were 75 metabolites that differentiated cowpea, 121 that differentiated pigeon pea, and 185 that differentiated common bean after post-hoc analysis (Fig. 1D).

The 75 metabolites that differentiated cowpeas from the other legumes include 45 lipids and lipid-like molecules, 22 unclassified metabolites, 3 organoheterocyclic compounds, 2 organic oxygen compounds, 2 phenylpropanoids and polyketides, and 1 organic acid or derivative (Table 1, Supplementary Table 3). Most of the metabolites that differentiated cowpeas from the other legumes, were significantly higher in cowpeas than the other two legumes. A selection of metabolites according to significance by p-value included TG(16:0/16:1(9Z)/18:2(9Z,12Z)), C41H102N10O3S2, TG(16:0/18:1(9Z)/18:2(9Z,12Z))_2, 1-[(9Z)-octadecenyl]-3-[(9Z)-octadecenoyl]-*sn*-glycerol, and TG(18:2(9Z,12Z)/18:2(9Z,12Z)/20:1(11Z)). Apart from C41H102N10O3S2, these metabolites belong to the glycerolipid class. Based on comparative analysis of the metabolites that differentiated cowpea within the legumes and between the cowpea varieties (described in section 3.1.3), 3-(all-*trans*-nonaprenyl)benzene-1,2-diol, N-tetracosanoylphytosphingosine, and sitoindoside II warrant further investigation as indicators for cowpeas and represent possible markers detectable in people following consumption.

**Table 1:** Classification of metabolites that differentiate three legume types.

Superclass	Class	Number of differentiating metabolites		
		Cowpea	Pigeon Pea	Common Bean
Benzenoids	Benzene and substituted derivatives	0	1	2
Hydrocarbons	Unsaturated hydrocarbons	0	2	0
Lipids and lipid-like molecules	Fatty Acyls	2	7	14
	Glycerolipids	12	19	15
	Glycerophospholipids	24	29	36
	Prenol lipids	2	2	15
	Saccharolipids	0	0	2
	Sphingolipids	1	3	2
	Steroids and steroid derivatives	4	3	12
Organic acids and derivatives	Carboximidic acids and derivatives	0	0	7
	Carboxylic acids and derivatives	0	1	0
	Organic phosphoric acids and derivatives	0	0	1
	Peptidomimetics	1	0	0
Organic nitrogen compounds	Organonitrogen compounds	0	0	3
Organic oxygen compounds	Organooxygen compounds	2	1	4
Organoheterocyclic compounds	Azoles	0	0	1
	Benzopyrans	1	0	0
	Indolizidines	1	0	1
	Lactones	0	0	1
	Pyrrolidines	0	1	0
	Quinolizines	0	0	1
	Tetrapyrroles and derivatives	1	0	0
Phenylpropanoids and polyketides	Cinnamic acids and derivatives	1	5	0
	Linear 1,3-diarylpropanoids	1	0	0
	Macrolactams	0	0	1
	Macrolides and analogues	0	0	1
	Tannins	0	1	0
NA	NA	22	46	66
Total		75	121	185

There were 121 metabolites that differentiated pigeon pea from the other legumes. This list included 63 lipid and lipid-like molecules, 46 unclassified metabolites, 6 phenylpropanoids and polyketides, 2 hydrocarbons, 1 benzenoid, 1 organic acid or derivative, 1 organic oxygen compound, and 1 organoheterocyclic compound (Table 1, Supplementary Table 3). Most of the differentiating metabolites were significantly higher in pigeon pea than the other legumes and metabolites with higher significance according to lower p-values included proline betaine, PE(16:0/22:6(4Z,7Z,10Z,13Z,16Z,19Z)), C14H9NS12, 22:0-Glc-Sitosterol, and DG(18:3(6Z,9Z,12Z)/18:2(9Z,12Z)/0:0). These metabolites are classified to the carboxylic acid, glycerophospholipid, unclassified, steroid, and fatty acyl classes, respectively.

#### 3.1.2 Fold-change metabolite analysis between legume types expanded the identification of a diverse suite of chemical profile distinctions

**Fig. 2:**
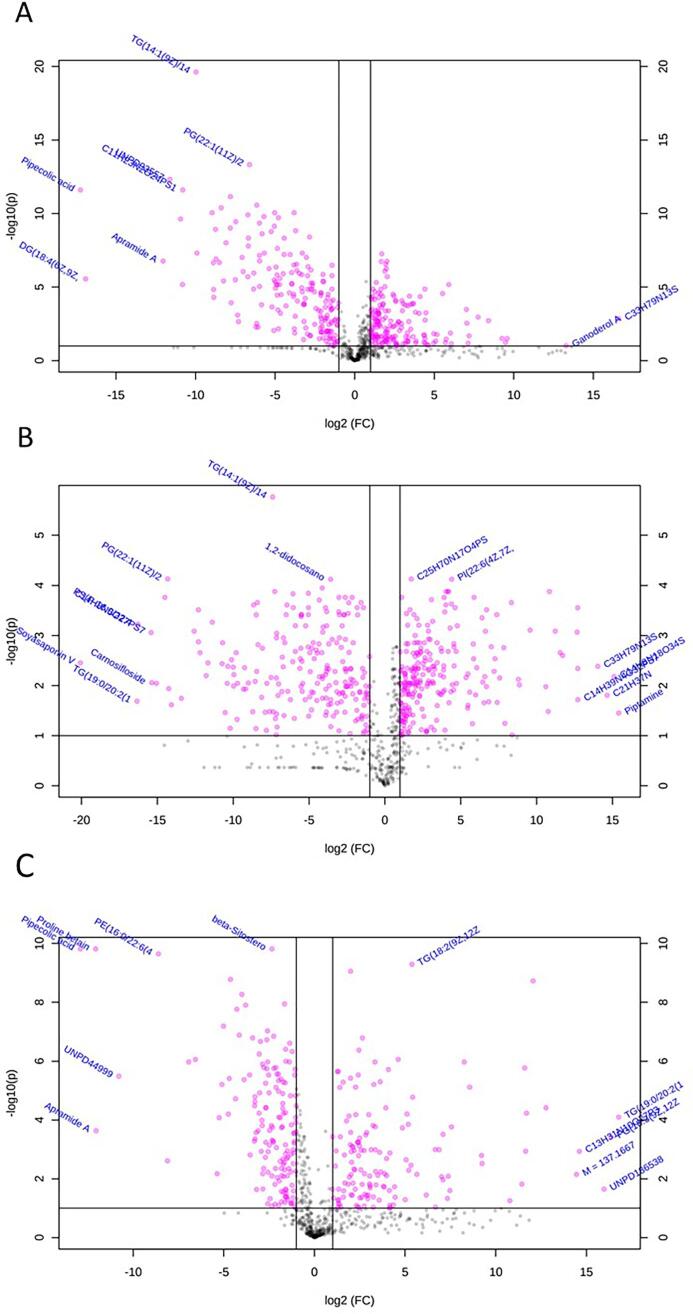
Volcano plots illustrating compound differences between two legume types. (A) cowpea versus common bean fold change volcano plot. (B) pigeon pea versus common bean fold change volcano plot. (C) cowpea versus pigeon pea fold change volcano plot. Metabolites with positive log2(fold change) values are higher in the first group of the comparison. Metabolites with negative log2(fold change) values are higher in the second group of the comparison.

Fold change analysis of the metabolites identified on Platform 1 revealed notable differences between cowpea and common bean. 173 metabolites were significantly higher in abundance in cowpea than in common bean, 183 were significantly higher in common bean than in cowpea, and 419 were not significantly different (Fig. 2). The most extreme log2(fold change) values observed in either direction were −17.235 and 16.594 for pipecolic acid and C33H79N13S, respectively (Supplementary Table 4). Pipecolic acid was one of the most significant metabolites found by ANOVA analysis (section 3.1.2). Post-hoc and fold change analyses both confirm that it is higher in abundance in common bean than the other two legumes.

Comparing fold-change differences in metabolite abundance between pigeon pea and common bean on Platform 1, revealed 233 metabolites significantly higher in pigeon pea than in common bean, 216 metabolites significantly higher in common bean than in pigeon pea, and 326 metabolites which did not show significant fold change differences (Fig. 2). The extreme log2(fold change) values observed in either direction were −20.07 and 15.416 for soyasaponin V and piptamine, respectively (Supplementary Table 4). Soyasaponin V was significantly higher in common bean and did not have a significant difference in means between cowpea and pigeon pea. Piptamine was significantly higher in pigeon pea than both cowpea and common bean and may warrant further investigation as a metabolite specific to pigeon pea.

Cowpea and pigeon pea fold change comparisons from Platform 1 revealed that 142 metabolites were significantly higher in cowpea than in pigeon pea, 154 were significantly higher in pigeon pea than in cowpea, and 479 did not show a significant fold change (Fig. 2). The extreme log2(fold change) values observed were −12.909 and 16.783 for pipecolic acid and TG(19:0/20:2(11Z,14Z)/20:4(5Z,8Z,11Z,14Z))[iso6], respectively (Supplementary Table 4). Pipecolic acid was a highly significant metabolite by ANOVA analysis (section 3.1.2), and was higher in common bean than the other two legumes. Post-hoc and fold change analysis resulted in the establishment of major metabolite differences between cowpea and pigeon pea.

#### 3.1.3 Key varietal differences in cowpea metabolite composition for varieties Dagbantuya, Sangyi, and Tukara

**Fig. 3:**
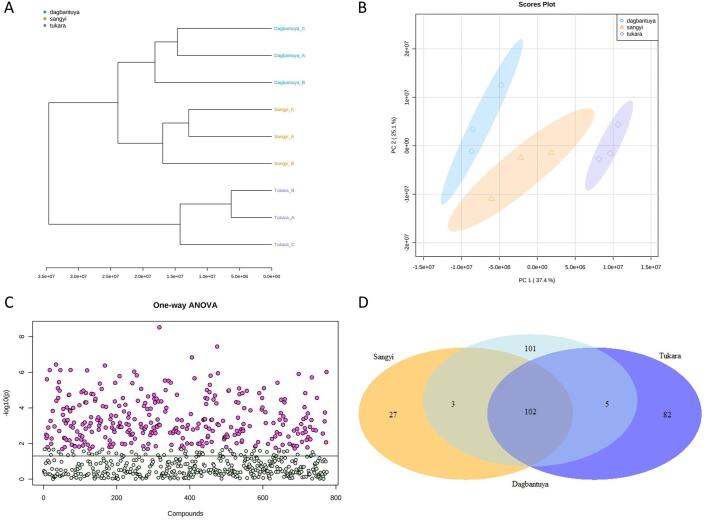
Cowpea variety metabolomes examined together by (A) Hierarchal cluster dendrogram based on Euclidean distance and Ward clustering. (B) Principal Component Analysis (PCA) scores plot of cowpea variety data. (C) One-way ANOVA plotting -log10(*p*-values) of all detected metabolites. Metabolites with significant differences in means across cowpea variety are plotted in pink (n = 320), metabolites with no significant difference in mean across cowpea variety are plotted in grey (n = 455). (D) Venn diagram indicating the number of metabolites that differentiate cowpea varieties based on Fisher’s least significant difference (LSD) post-hoc analysis.

Comparing the metabolic profiles of three cowpea varieties (Dagbantuya, Sangyi, and Tukara) with principal component analysis (PCA) had similar observations in metabolite differences identified with the metabolite-based dendrogram in Fig. 3A and B). Both plots indicated that Dagbantuya and Sangyi are more similar to each other than they are to Tukara.

The one-way ANOVA testing supported 320 metabolites with significant differences in means, out of the 775 detected from Platform 1 (Fig. 3C). Of those, 101 metabolites differentiated Dagbantuya, 27 differentiated Sangyi, and 82 metabolites differentiated Tukara after post-hoc analysis (Fig. 3D). Cowpea varietal comparisons bring an intentional highlight on the following compounds C18H34N6O6S16, C45H106N12O3S2, C14H47N4O32PS12, 1,2-Di-(9Z,12Z,15Z-octadecatrienoyl)-3-(Galactosyl-alpha-1–6-Galactosyl-beta-1)-glycerol, and uvarigrin;(+)-uvarigrin based on ANOVA *p*-values. With the exception of 1,2-Di-(9Z,12Z,15Z-octadecatrienoyl)-3-(Galactosyl-alpha-1–6-Galactosyl-beta-1)-glycerol, the other metabolites were all higher in Dagbantuya than the other two cowpea varieties (Supplementary Table 3).

The 101 metabolites that differentiated Dagbantuya from the other cowpeas on Platform 1 include 56 lipids and lipid-like molecules, 40 unclassified metabolites, 3 organic oxygen compounds, 1 organic acid or derivative, and 1 phenylpropanoid and polyketide (Table 2). 73 of these 101 metabolites (equivalent to 72.3%) that differentiated Dagbantuya from the other varieties were higher in abundance in Dagbantuya than Sangyi and Tukara. The 27 metabolites that differentiated Sangyi from the other two cowpeas on Platform 1 include 18 lipids and lipid-like molecules, 4 unclassified metabolites, 2 benzenoids, 1 nucleoside/nucleotide/analogue, 1 organic acid or derivative, and 1 phenylpropanoid and polyketide (Table 2). 14 of these 27 metabolites (equivalent to 51.9%) differentiating Sangyi from the other varieties were lower in abundance in Sangyi than the other varieties. Lastly the 82 metabolites that differentiated Tukara from the other cowpeas on Platform 1 include 48 lipids and lipid-like molecules, 25 unclassified metabolites, 4 organic oxygen compounds, 3 organic acids and derivatives, and 2 organoheterocyclic compounds (Table 2). 64 of these 82 metabolites (equivalent to 78.0%) differentiating Tukara from the other varieties were higher in abundance in Tukara than the other varieties. Details can be found in Supplementary Table 3.

**Table 2:** Classification of metabolites that differentiate three cowpea varieties.

Superclass	Class	Number of differentiating metabolites		
		Dagbantuya	Sangyi	Tukara
Benzenoids	Benzene and substituted derivatives	0	2	0
Lipids and lipid-like molecules	Fatty Acyls	11	3	6
	Glycerolipids	13	6	13
	Glycerophospholipids	16	5	23
	Prenol lipids	4	1	3
	Sphingolipids	2	0	1
	Steroids and steroid derivatives	10	3	2
Nucleosides, nucleotides, and analogues	Purine nucleosides	0	1	0
Organic acids and derivatives	Carboximidic acids and derivatives	0	0	1
	Carboxylic acids and derivatives	1	1	1
	Organic phosphoric acids and derivatives	0	0	1
Organic oxygen compounds	Organooxygen compounds	3	0	4
Organoheterocyclic compounds	Quinolines and derivatives	0	0	1
	Tetrapyrroles and derivatives	0	0	1
Phenylpropanoids and polyketides	Cinnamic acids and derivatives	1	0	0
	Linear 1,3-diarylpropanoids	0	1	0
NA	NA	40	4	25
Total		101	27	82

### 3.2 Comprehensive metabolic pathway coverage for cowpea and pigeon pea using two metabolomics platforms

The types of compounds detected and identified from Platform 1 and 2 in this study provided breadth and depth to the measurable differences in composition between cowpea and pigeon pea. The results from Platform 2 were distinct from the metabolite lists that differentiate cowpea varieties from pigeon pea in Platform 1. There were 49 metabolites common to all cowpea varieties, and 337 common to both cowpea and pigeon pea on Platform 2. By cowpea variety, Platform 2 did not detect metabolites unique to Dagbantuya or Tukara, but pheophytin A was uniquely detected for Sangyi. There were eight metabolites detected uniquely to pigeon pea (Adua), although none of these were detected on Platform 1. Both Platform 1 and 2 were used to create a list of unique metabolites that may have utility as food chemical markers and dietary exposure biomarkers of intake in people with further validation. Both platforms provided unique strengths and helped overcome the limitations of a single platform. Given that the number of detected metabolites and annotations differed across platforms, we also leveraged the opportunity for increased sensitivity to measure abundances using distinct compound annotation software.

**Fig. 4:**
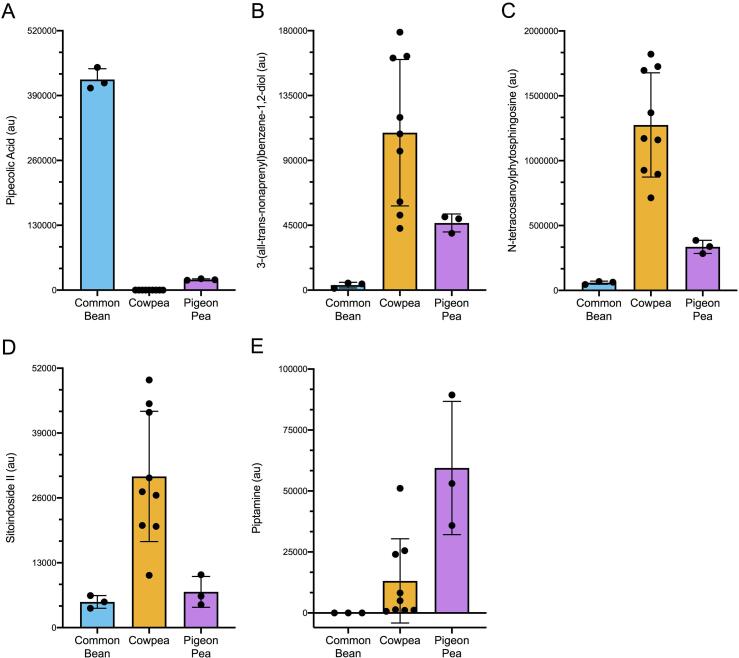
Median scaled relative abundance for metabolites distinguishing cowpeas, common bean and pigeon pea using Platform 1. Metabolites for (A) all legume types, (B-D) cowpea, (E) pigeon pea. Metabolite abundances are in arbitrary relative abundance units (au).

Platform 1 consistently detected pipecolic acid (*p*-value = 1.22E-16) as differentiating between legume types, whether by ANOVA or fold change analysis. Although pipecolic acid had higher abundance in common bean, pipecolic acid results support that it is common metabolite across all legume types analyzed in this study. The relative abundance comparisons are visualized in Fig. 4.

Metabolites that differentiated cowpea from the other legume types were identified by Platform 1, since this analysis compared cowpea to both pigeon pea and common bean. To narrow down the list of cowpea metabolites to focus on, we looked for metabolites that were common between both the legume analysis (described in section 3.1.1), as well as the cowpea varietal analysis (described in section 3.1.3) and put an intentional focus on metabolites that were higher in cowpea than the other legumes. We identified 3-(all-*trans*-nonaprenyl)benzene-1,2-diol (*p*-value = 0.0057), N-tetracosanoylphytosphingosine (*p*-value = 0.0002), and sitoindoside II (*p*-value = 0.0039), which may be identifiers of cowpea consumption. These metabolites were all higher in cowpea than the other two legumes (Fig. 4).

For cowpea varietal comparisons, Platform 2 did not result in identification of metabolites unique to Dagbantuya, which provided rationale to focus on Platform 1 for this variety. Based on the ANOVA, post-hoc differentiation, and reports in the literature, tonkinelin (*p*-value = 3.25E-05) is considered unique to the cowpea variety Dagbantuya. Platform 2 analysis did result in metabolite identification of pheophytin A as unique to the cowpea variety Sangyi. Due to its detection in one variety only and based on Platform 2 having a higher level of annotation confidence, pheophytin A was specifically designated to the cowpea variety Sangyi. Both Platform 1 and 2 detected the compound linoleoyl ethanolamide and for distinguished abundance by variety. Platform 1 showed that linoleoyl ethanolamide was significantly lower in Tukara than in Dagbantuya and Sangyi (*p*-value = 0.0045), and Platform 2 only detected the compound in Dagbantuya and Sangyi. Because important metabolites can also be low in abundance, or absent, linoleoyl ethanolamide warrants further investigation as a metabolite related to consumption of Tukara.

Relating to pigeon pea, Platform 1 detected piptamine (*p*-value = 0.0031) as significant and differentiating from cowpea and common bean (Fig. 4). Platform 2 detected eight compounds unique to pigeon pea, including tryptophan betaine, chiro-inositol, phenylacetylglutamate, gamma-glutamyl-GABA, N,N-dimethylalanine, as well as three unnamed compounds (Supplementary Table 2). Based on the ANOVA significance, post-hoc differentiation, and fold change of piptamine on Platform 1, this compound is highlighted for further investigation as a metabolite that may be indicate pigeon pea consumption when compared to other cowpeas or beans.

## 4 Discussion

Legumes contain a diverse and beneficial range of chemical compounds with many established health and nutritional benefits. The metabolic profiles of different legume types in fully cooked forms that are safe for consumption by people are limited. This study applied an intentional focus on the comparative analysis of local cowpea varieties and pigeon pea commonly consumed by households in Ghana. Two separate metabolite detection and analysis workflows supported the generation of novel compound lists for cooked cowpea and pigeon pea. The two platforms had different food chemical extraction reagents and elution techniques applied to garner a comprehensive chemical profile. Different extraction and elution methods alongside the column separation and detection platforms allowed for highly specialized distinctions in chemical identity to be revealed for these legumes and with broader coverage of metabolic pathways and chemical sub-classes, namely for lipids and amino acids. The results demonstrated the increased capacity to decipher metabolite relationships between legumes and between cowpea varieties. This study shows strong support for the cowpea metabolite profile as more similar to that of common bean than it is to the pigeon pea. Notably, these findings mirror the phylogenetic relationships that have been previously shown (Ji et al., 2019). Importantly, these results support a novel finding that the cowpea Dagbantuya and Sangyi metabolite profiles are more similar to each other than to the Tukara cowpea variety.

Metabolomics is an incredibly informative tool for food composition profiling and can aid plant breeding as well as nutrition studies. The identification of food chemical biomarkers for use in dietary exposure assessments from consumption relates to the growing needs in assessing compliance to feeding interventions and without the bias of self-reported data (Hedrick et al., 2012). Advancing our knowledge for which food metabolites are present in staple legume-based diets is relevant to future understanding of their bioactivity in the gut. These bioactive molecules identified herein may also guide and inform crop breeding efforts aimed at either increasing, maintaining, or reducing phytochemicals that have important nutritional and agronomic traits. Metabolomics-assisted breeding is a functional screening approach to select for desired phenotypes early in the breeding process (Fernie & Schauer, 2009). This report shows that cowpeas and pigeon pea contain metabolites such as 3-(all-*trans*-nonaprenyl)benzene-1,2-diol, N-tetracosanoylphytosphingosine, sitoindoside II, and piptamine, which have many established health benefits and represent promising targets for breeding programs within West Africa and as well as geographically distinct regions.

This study identified three novel cowpea metabolites; 3-(all-*trans*-nonaprenyl)benzene-1,2-diol, N-tetracosanoylphytosphingosine, and sitoindoside II, that have published literature suggesting associations with human health benefits (Bentinger et al., 2010, Dahlén and Pascher, 1972, Poon et al., 1999, Satmbekova et al., 2018). The 3-(all-*trans*-nonaprenyl)benzene-1,2-diol is a prenol lipid that plays a role in *E. coli* Coenzyme Q biosynthesis (Poon et al., 1999). Coenzyme Q has well established anti-inflammatory properties (Bentinger et al., 2010). N-tetracosanoylphytosphingosine is a sphingolipid, which exhibits immunological activity (Dahlén & Pascher, 1972). Sitoindoside II is a steroid/steroid derivative that is found in the plant *Cichorium intybus L.*, often used in traditional medicine for its diuretic, anti-inflammatory, cardiotonic, liver tonic, and digestive benefits (Satmbekova et al., 2018). These metabolites merit recognition within cowpea breeding programs and for examination as candidate dietary exposure biomarkers in people after regular cowpea consumption.

Novel cowpea metabolites of varietal distinction from this study include tonkinelin, pheophytin A, and linoleoyl ethanolamide, for Dagbantuya, Sangyi, and Tukara, respectively. These components have not been previously reported from cowpea, but information is known from other plants and cellular function studies with *in vitro* assays. Tonkinelin is a fatty acyl that has been identified in *Uvaria tonkinensis* and has established acetogenic effects (Chen & Yu, 1996). Pheophytin A is involved in chlorophyll metabolism and contributes to dark pigment colors (Yilmaz & Gökmen, 2015). Pheophytin A metabolite that contributes to pigmentation. . Sangyi has the darkest pigmentation of the cowpea varieties analyzed. Linoleoyl ethanolamide is a carboximidic acid/derivative that has anti-inflammatory effects (Ishida et al., 2013).

The pigeon pea analysis highlighted the benezenoid metabolites piptamine, and phenylacetylglutamate, as well as the amino acid derivative N,N-dimethylalanine. Piptamine is a known antibiotic, first isolated from *Piptoporus betulinus* (Schlegel, Luhmann, Hartl, & Grafe, 2000). The bioactivity for this compound when delivered from food remains unclear and merits further investigation for impact by post-harvest and processing conditions. Phenylacetylglutamate has been recognized as a uremic retention product and although there is little evidence for this compound from pigeon pea to have this direct biological effect, one study was able to demonstrate an anti-proliferative effect on cancer cells under certain conditions (Vanholder, Pletinck, Schepers, & Glorieux, 2018). N,N-dimethylalanine is another pigeon pea metabolite that has not been extensively characterized, but this compound had been previously investigated to characterize the mechanism in which neoagarotetraose alleviates intestinal inflammation. The N,N-dimethylalanine pathway was suggested to play a role in the prebiotic actions of neoagarotetraose (Liu et al., 2020). We put forth that pigeon pea derived metabolites in the diet do merit evaluation for actions in the human gut.

Legumes and particularly cowpeas can help alleviate malnutrition and support healthy growth of children in low-income countries where cowpeas are prevalent and well adapted for local climates (Stephenson et al., 2017). These cooked legume flours analyzed herein have been fed in controlled doses to children and pregnant women from Ghana. The studies supported feasibility of increasing cowpea doses over a period of 20-days and ongoing analysis will guide dietary exposure biomarker identifications and assessments using the blood and urine that were collected. This study substantially contributes to the food metabolite profile analysis of cowpea and seeks to link findings with promising agronomic traits, nutritional value, and preferential consumption observed by local communities in Sub-saharan Africa (Abizari et al., 2013, Jayathilake et al., 2018).

Study limitations exist for this non-targeted metabolic profiling approach. In particular, metabolite identification and annotation can vary across platforms due to differences in instrument sensitivity and software. The majority of metabolites identified using both platforms, were annotated and classified though RAMClustR, MSFinder and in-house libraries, supporting the premise that computational annotation tools can provide valuable insight in the absence of spectral libraries. Targeted quantification of the metabolite levels using internal standards would give useful information on the absolute quantities of compounds available after consumption. Integrating data sets from this study with additional cowpea varieties and from biospecimens collected after legume consumption in people may improve our knowledge of bioactivity alongside identification of relevant chemical biomarkers.

## 5 Conclusions

This study supports that the analysis of metabolic profiles from cooked flours of three different cowpea varieties and pigeon pea commonly consumed in Ghana had strong capacity to identify novel components and distinguish the food chemical profile from a reference legume (common bean). The use of two distinct metabolite extractions and profiling workflows bolstered our knowledge base of metabolite relationships between cowpea, pigeon pea, and common bean that mirrored genetics. The metabolite profiles of cowpea varieties Dagbantuya and Sangyi were considered to be more similar to each other than the variety, Tukara based on metabolite abundance and overlapping identifications. This study identified several novel metabolites associated with each legume species and cowpea variety, including Dagbantuya (tonkinelin), Sangyi (pheophytin A), Tukara (linoleoyl ethanolamide), and pigeon pea (piptamine). These findings have advanced our knowledge of the food chemical profiles provided from human dietary intake of cowpea and pigeon pea because the analysis was conducted used fully cooked forms. Future work to quantify and validate the metabolites for differentiating legumes in the diet is needed alongside an integrated biomarker analysis from feeding studies with these foods. The utility of food specific analysis is to support ongoing breeding strategies geared towards improving nutrition and food function.

## Declaration of Competing Interest

The authors declare that they have no known competing financial interests or personal relationships that could have appeared to influence the work reported in this paper.
